# Development and Evaluation of Mouth Dissolving Films of Amlodipine Besylate for Enhanced Therapeutic Efficacy

**DOI:** 10.1155/2014/520949

**Published:** 2014-03-27

**Authors:** K. M. Maheswari, Pavan Kumar Devineni, Sravanthi Deekonda, Salma Shaik, Naga Pravallika Uppala, Buchi N. Nalluri

**Affiliations:** Department of Pharmaceutics, KVSR Siddhartha College of Pharmaceutical Sciences, Vijayawada, Andhra Pradesh 520010, India

## Abstract

The present investigation was undertaken with an objective of formulating mouth dissolving films (MDFs) of Amlodipine Besylate (AMLO) to enhance convenience and compliance of the elderly and pediatric patients for better therapeutic efficacy. Film formers like hydroxy propyl methyl cellulose (HPMC) and methyl cellulose (MC) along with film modifiers like poly vinyl pyrrolidone K30 (PVP K30), and sodium lauryl sulphate (SLS) as solubilizing agents were evaluated. The prepared MDFs were evaluated for *in vitro* dissolution characteristics, *in vitro* disintegration time, and their physicomechanical properties. All the prepared MDFs showed good mechanical properties like tensile strength, folding endurance, and % elongation. MDFs were evaluated by means of FTIR, SEM, and X-RD studies. MDFs with 7.5% (w/w) of HPMC E3 gave better dissolution properties when compared to HPMC E5, HPMC E15, and MC. MDFs with PVP K30 and SLS gave superior dissolution properties when compared to MDFs without PVP K30 and SLS. The dissolution properties of MDFs with PVP K30 were superior when compared to MDFs with SLS. In the case of F3 containing 7.5% of HPMC E3 and 0.04% of PVP K30, complete and faster release was observed within 60 sec when compared to other formulations. Release kinetics data reveals diffusion is the release mechanism.

## 1. Introduction

The oral cavity has been the most prominent site of drug delivery for a long period of time. In 1847, Sobrero found that nitroglycerine was absorbed from the oral cavity [1]. Since then various active substances have been investigated for local or systemic use. Recent developments in the formulation technology have presented viable dosage alternatives from the oral route for pediatrics, geriatric, bedridden, nauseous, or noncompliant patients. Novel bioadhesive mucosal dosage forms including adhesive tablets, gels, patches, and more recently the use of polymeric films for oral cavity delivery, also known as MDFs, gained attention in formulation research. MDFs, a new and novel drug delivery system for per oral delivery of the drugs, were developed based on the technology of the transdermal patch [2]. The delivery system consists of a very thin oral strip, which is simply placed on the patient's tongue or any oral mucosal tissue; instantly wet by saliva the film rapidly hydrates and adheres onto the site of application. It then rapidly disintegrates and dissolves to release the medication for oromucosal absorption. Various film formers like polyvinyl alcohol, PVP, maltodextrin, HPMC, hydroxy propyl cellulose (HPC), MC, sodium carboxy methyl cellulose (Na CMC), chitosan, and some natural gums have been used in the production of films [3].

AMLO is a long acting second generation dihydro calcium channel blocker with actions similar to nifedipine used in the management of hypertension and angina pectoris [4]. In hypertension, the usual initial dose is 5 mg daily, increased if necessary to 10 mg once daily. It is well absorbed following oral administration with peak blood concentration occurring after 6–12 hours. Elimination from the plasma is biphasic with a terminal elimination half-life of about 30 to 50 h. Absolute bioavailability has been estimated to be between 60 and 65% [5]. Few reports were published on the mouth disintegrating tablets of AMLO [6–10]. Presently, AMLO is marketed in the form of ODTs (Norvasc) and IR tablets (Amlogard, Acord, Stamlo, etc.). However, ODTs are associated with a disadvantage of grittiness in the mouth. Keeping in view with patient compliance and need of better therapeutic efficacy and since no research work has been done on AMLO MDFs, the present investigation was aimed at preparation and evaluation of AMLO MDFs to ensure quick onset of action.

## 2. Material and Methods

### 2.1. Materials

AMLO, pine apple flavor and Aspartame were obtained from Darwin Laboratories, Vijayawada. HPMC E3, E5, E15, and MC were obtained from Colorcon Asia Ltd., India. Methanol, SLS, and PVP K30 were purchased from Loba Chemie, Mumbai. All other reagents of analytical grade were used.

### 2.2. Preparation of Artificial Saliva

Artificial saliva was prepared as follows [11]: sodium chloride: 0.844 g; potassium chloride: 1.2 g; calcium chloride dehydrate: 0.193 g; magnesium chloride hexahydrate: 0.111 g; and potassium phosphate dibasic: 0.342 g. These ingredients were added one by one to 500 mL of distilled water and then the volume was made up to 1000 mL using water. The pH was adjusted with 0.1 N hydrochloric acid to 5.7.

### 2.3. Preparation of AMLO MDFs

AMLO MDFs were prepared as per formula given in Table 1 to a batch size of 5 g. Drug was dissolved in a mixture of solvents (water and methanol) in a beaker and other ingredients were added one by one and finally polymer HPMC/MC was added and mixed thoroughly and the mixture was sonicated for 5 min to remove entrapped air bubbles and casted on a glass plate with a wet film applicator set at 30 mil thickness (750 *μ*m) and it was dried at 40°C for 60 min in hot oven air. Then the dried films were peeled off from the glass plate, cut into appropriate sizes, and stored in desiccator until use. The films were evaluated for the following properties.

### 2.4. Drug-Excipient Compatibility Studies

#### 2.4.1. FTIR Studies

Samples were analyzed using an ATR-FTIR spectrometer (Bruker, Germany). ATR spectra were measured over the wave number range of 4000–500 cm^−1^ at a resolution of 1.0 cm^−1^. The powder or film sample was simply placed onto the ATR crystal and the sample spectrum was collected.

#### 2.4.2. X-RD Analysis

Samples were analyzed using Bruker D8, advanced diffractometer (Bruker-AXS Karl Sruhe, Germany) using cu-k*α* X-radiation (*λ* = 1.54060 Å) at 45 kV and 40 mA power. X-ray diffraction patterns were collected over the 2*θ* range of 4–44° at a scan rate of 1°/min. The position and intensities of diffraction peaks were considered for the identification of AMLO in different samples.

#### 2.4.3. SEM Analysis

The morphology and surface topography of the film were examined by scanning electron microscopy (SEM-JEOL, JSM-840A, Japan). The samples to be examined were mounted on the SEM sample stab using a double-sided adhesive tape. The samples mounted were coated with gold (200 Å) under reduced pressure (0.001 torr) for 5 min to improve the conductivity using an Ion sputtering device (JEOL, JFC-1100 E, Japan).

### 2.5. Evaluation Parameters for AMLO MDFs

#### 2.5.1. Morphological Properties

Properties such as homogeneity, color, transparency, and surface of AMLO MDFs were tested visually. All the formulations were stored at room temperature (25 ± 3°C) with relative humidity of approximately 65 ± 5% and were tested periodically every month for a period of 6 months. The films were packed in aluminum foil pouches.

#### 2.5.2. Drug Content

One cm^2^ film was taken in a 10 mL volumetric flask and dissolved in 5 mL of methanol and then final volume was made up with methanol. Samples were suitably diluted with artificial saliva and the absorbance was measured at 238 nm. The estimations were carried out in triplicate.

#### 2.5.3. Variation of Mass

Mass of 1 cm^2^ film from different batches of the formulations was noted on electronic balance. The estimations were carried out in triplicate.

#### 2.5.4. Thickness

The thickness of film was evaluated using a screw gauge with a range of 0–10 mm and revolution 0.001 mm. Anvil of the thickness gauge was turned and the film was inserted after making sure that the pointer was set to zero. The film was held on the anvil and the reading on the dial was noted down. The estimations were carried out in triplicate.

#### 2.5.5. *In Vitro* Disintegration Studies

Disintegration time gives an indication about the disintegration characteristics and dissolution characteristics of the film. In case of MDFs the disintegration and dissolution procedures are hardly distinguishable. If the MDF disintegrates it concurrently dissolves in a small amount of saliva which makes it difficult to mimic these natural conditions and measures with an adequate method. However, in the present investigation two methods of disintegration were adopted.

*Drop Method*. In the first method one drop of distilled water was dropped by a pipette onto the oral films. The films were placed on a glass slide and then the glass slide was placed planar on a petridish. The time until the film dissolved and caused a hole within the film was measured. The estimations were carried out in triplicate.

*Petridish Method*. In this method 2 mL of distilled water was placed in a petridish and one film was added on the surface of the water and the time required until the oral film dissolved completely was measured. Drug-loaded films were investigated under both methods. The estimations were carried out in triplicate.

#### 2.5.6. Tensile Strength

Tensile strength is the maximum stress applied to a point at which the film specimen breaks [12]. It is calculated by the load at rupture divided by the cross-sectional area of the film as given below:

(1) $$\begin{array}{c} \text{Tensile strength}=\frac{\text{load at failure}\times 100}{\text{film thickness}\times \text{film width}}. \end{array}$$

It was measured using Shimadzu AG-100kNG (Winsoft tensile and compression testing). The film of size 3 × 2 cm^2^ and free of physical imperfections was placed between two clamps held 10 mm apart. The film was pulled by a clamp at a rate of 5 mm/min. The whole experiment was carried out in triplicate.

#### 2.5.7. Percent Elongation (% E)

When stress is applied the film sample stretches and is referred to as strain. Strain is basically the deformation of the film divided by the original dimension of the film. Generally elongation of the film increases as the plasticizer concentration increases [13].

Percentage elongation was calculated by measuring the increase in length of the film after tensile strength measurement by using the following formula;

(2) $$\begin{array}{c} \text{Percentage elongation}=\frac{\left[L-L_{0}\right]\times 100}{L_{0}}, \end{array}$$

where *L* = final length and *L*_0_ = initial length.

The estimations were carried out in triplicate.

#### 2.5.8. Young's Modulus

Young's modulus or elastic modulus is the measure of stiffness of film. It is represented as the ratio of applied stress over strain in the region of elastic deformation as follows:

(3) $$\begin{array}{c} \text{Young's modulus}=\frac{\text{slope}\times 100}{\text{Film thickness}\times \text{cross head speed}}. \end{array}$$

Hard and brittle film demonstrates a high tensile strength and Young's modulus with small elongation. The estimations were carried out in triplicate.

#### 2.5.9. Folding Endurance

Folding endurance was determined by repeated folding of the film at the same place till the film breaks. This gives an indication of the brittleness of the film. The number of times the film was folded without breaking was computed as the folding endurance value [14]. The estimations were carried out in triplicate.

#### 2.5.10. *In Vitro* Dissolution Studies

**Figure 1:**
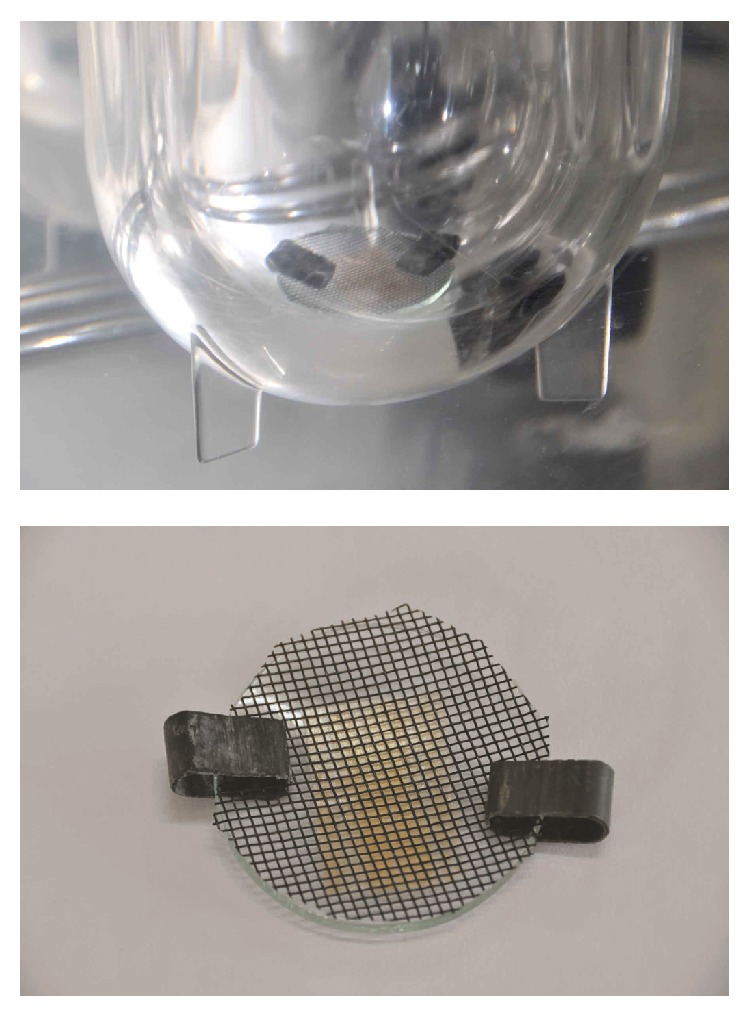
Dissolution setup assembly.

The* in vitro* dissolution studies were conducted using 500 mL of artificial saliva as dissolution medium with modified type 5 dissolution apparatus. A temperature of 37°C and 50 rpm was used. Each film with a dimension of appropriate size equivalent to 5 mg of AMLO was placed on a watch glass covered with nylon wire mesh as shown in Figure 1. The watch glass was then dropped into a dissolution flask. Five mL samples were withdrawn at 10, 20, 30, 40, 50, 60, 80, 100, and 120 sec time intervals and every time replaced with 5 mL of fresh dissolution medium. The samples were analyzed by measuring absorbance at 238 nm. The dissolution experiments were conducted in triplicate.

## 3. Results and Discussion

### 3.1. Preparation and Physical Characterization of AMLO MDFs

Initially placebo MDFs were prepared with different polymers like HPMC (E3, E5, E15, E50, and K4 M), HPC, MC, NaCMC, PVP, gelatin, Polyox, and sodium alginate. Finally, from these trials and results obtained, HPMC E3, HPMC E5, HPMC E15, HPMC E50, MC, and NaCMC were selected for further development. Appropriate quantity of AMLO equivalent to 100 mg of Amlodipine base was added to the formulation and the MDFs were prepared. Crystallization of the drug was observed over a period of time with polymers HPMC E50 and NaCMC. Hence, these two polymers were excluded from the study. Different AMLO MDFs were prepared using HPMC E3, HPMC E5, HPMC E15, and MC as per the formulae given in Table 1. In total, a 5 g batch size of formulation gave approximately 130 cm^2^ film area.

### 3.2. FTIR Studies

**Figure 2:**
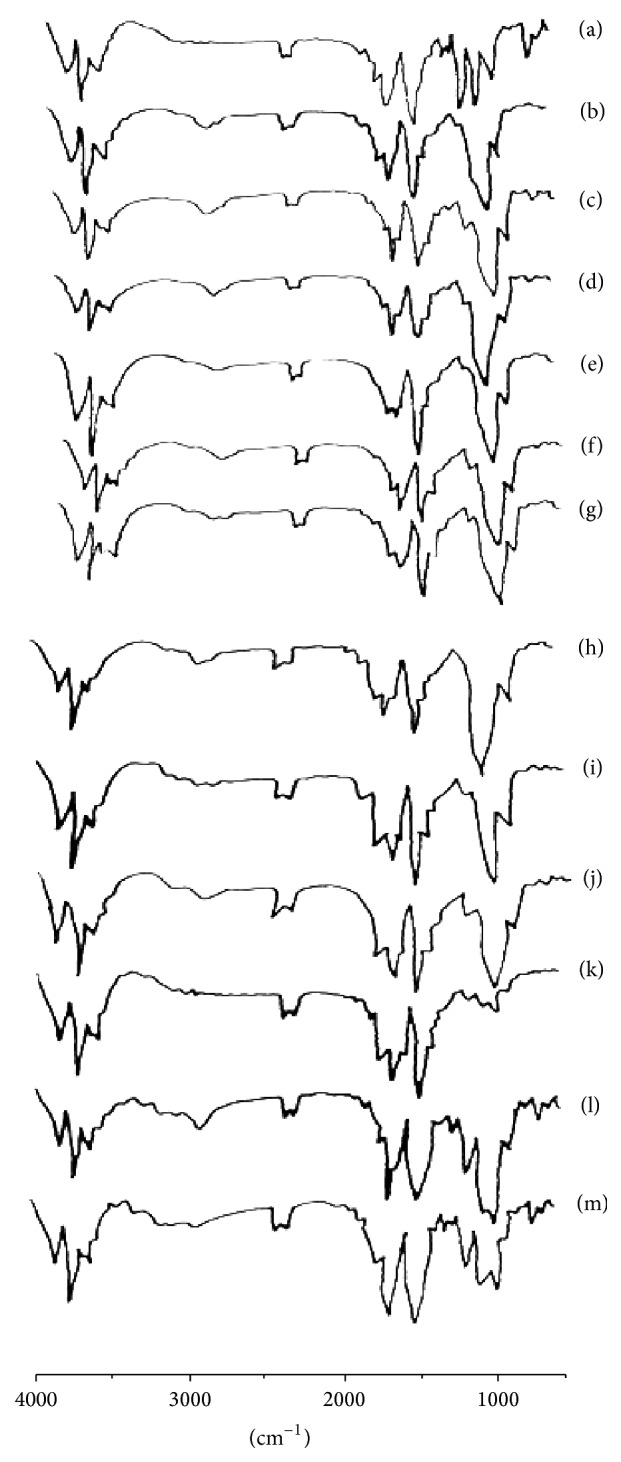
FTIR spectra of pure AMLO (a); AMLO + HPMC E3 film (b); AMLO + HPMC E3 + SLS film (c); AMLO + HPMC E3 + PVP film (d); AMLO + HPMC E5 film (e); AMLO + HPMC E5 + SLS film (f); AMLO + HPMC E5 + PVP film (g); AMLO + HPMC E15 film (h); AMLO + HPMC E15 + SLS film (i); AMLO + HPMC E15 + PVP film (j); AMLO + MC film (k); AMLO + MC + SLS film (l); and AMLO + MC + PVP film (m).

Pure AMLO showed IR absorption bands at 1298 cm^−1^ for the ethyl ester, 1197 cm^−1^ for sulfonic acid salts, 1116 cm^−1^ for the aliphatic ethers, and 2889 cm^−1^ for the stretching vibration of N–H bond in the dihydropyridine ring. These characteristic IR absorption bands of AMLO were all retained in the MDFs. These results indicate that there is no interaction between the AMLO and excipients in the MDFs. The spectra were shown in Figure 2.

### 3.3. X-RD Studies

**Figure 3:**
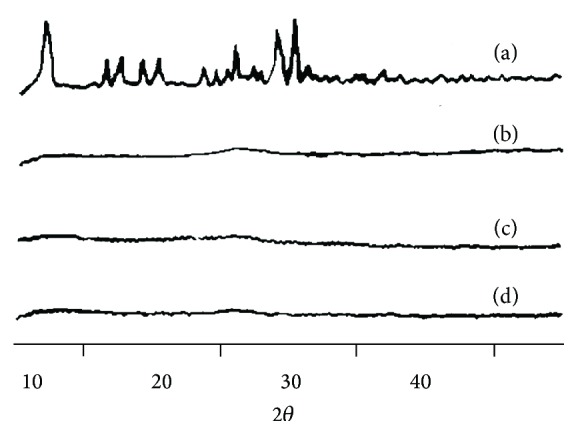
X-RD spectra of pure AMLO (a); AMLO + HPMC E3 film (b); AMLO + HPMC E3 + SLS film (c); and AMLO + HPMC E3 + PVP film (d).

Selected AMLO MDFs (F1, F2, and F3) were subjected to X-RD studies in order to investigate the crystallographic properties of AMLO in MDFs. AMLO showed characteristic peaks at 6.5°, 20.6°, 23.6°, and 24.93° 2*θ*. The X-ray diffractograms of the AMLO MDFs showed weak or no signals when compared to the characteristic peaks of pure AMLO. This may be due to molecular dispersion of AMLO within the MDFs. Overall, together with SEM data the X-RD results clearly indicate that the AMLO was not in crystalline state in MDFs. The X-ray diffractograms are shown in Figure 3.

### 3.4. SEM Analysis

**Figure 4:**
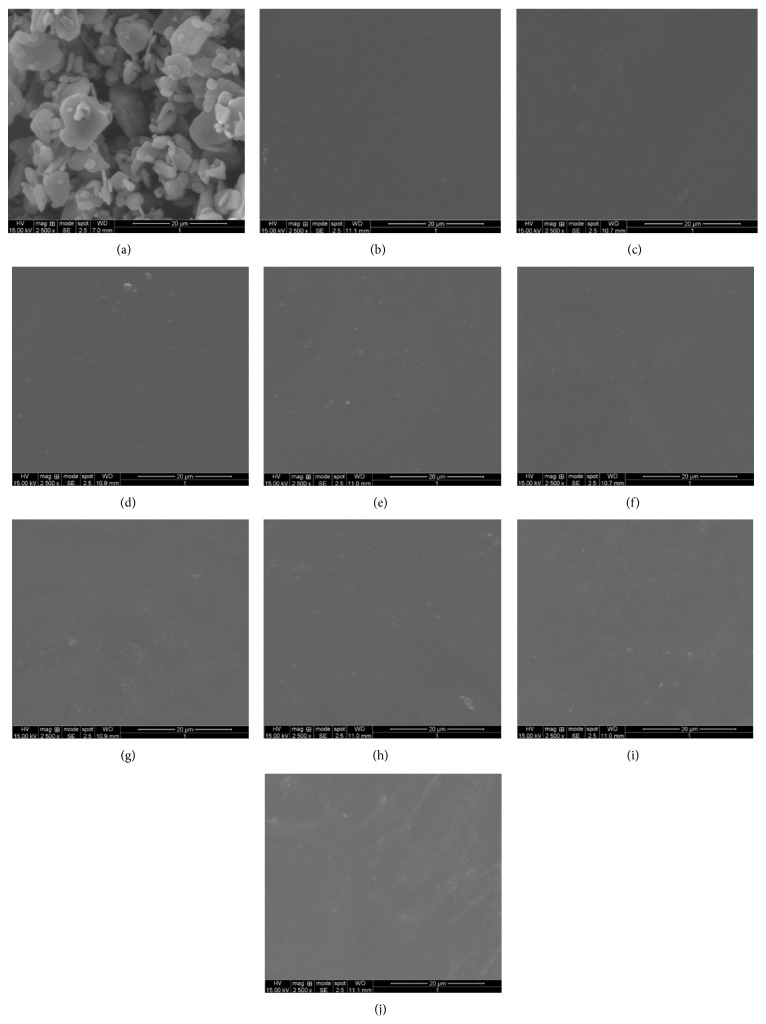
SEM photographs of pure AMLO (a); AMLO + HPMC E3 film (b); AMLO + HPMC E3 + SLS film (c); AMLO + HPMC E3 + PVP film (d); AMLO + HPMC E5 film (e); AMLO + HPMC E5 + SLS film (f); AMLO + HPMC E5 + PVP film (g); AMLO + HPMC E15 film (h); AMLO + HPMC E15 + SLS film (i); and AMLO + HPMC E15 + PVP film (j).

Macroscopically the prepared AMLO MDFs were clear and colorless. The scanning electron photomicrograph of the selected MDFs and also pure AMLO at 2500x magnification are shown in Figure 4. The SEM photographs of MDFs showed smooth surfaces without any scratches or transverse striations indicating that AMLO is uniformly distributed and no crystals of AMLO were observed in the MDFs.

### 3.5. Morphological Properties

AMLO MDFs were visually tested for homogeneity, transparency, color, and smoothness and results are given in Table 2. All the formulations showed no change in the properties at the end of the 6-month time period when compared to the initial properties and especially no crystallization of the AMLO was observed.

### 3.6. Drug Content

Films of 1 cm^2^ were cut from different places of the whole films and AMLO content was estimated. The results are given in Table 2. These results indicated a good uniformity of AMLO within films, and overall good solubilization of AMLO in the formulations was observed.

### 3.7. Variation of Mass

Films of 1 cm^2^ were cut from different batches and weighed. The results are given in Table 2. Same mass of film was obtained with three batches of films indicating reproducibility of preparation method and formulation.

### 3.8. Thickness

The thickness was measured with a screw gauge at different places of the MDFs in order to evaluate the reproducibility of the preparation method. Around 90% of wet film thickness was lost during drying. The results are given in Table 2 and a good uniformity of thickness was observed. MDFs with PVP and SLS showed an increase in the thickness of the film which in turn reflected increase in drug content and variation of mass compared to films without PVP and SLS Table 2.

### 3.9. Disintegration Time

The results of disintegration time are given in Table 2. These results indicated that the E3 formulations disintegrated faster than the E5, E15, and MC formulations. The AMLO MDFs with PVP disintegrated faster than the MDFs with and without SLS formulations. With the petri dish method F3, F6, and F9 formulations disintegrated/dissolved faster than the other formulations.

### 3.10. Tensile Strength, % Elongation, and Elastic Modulus

MDFs should possess moderate tensile strength, high % elongation (% E), low EM, and high percent of drug release. The results revealed that all the films showed moderate tensile strength values. Films of F1, F3, F6, and F9 showed highest % E when compared with other formulae and F3 has lowest EM when compared with other formulae. The results were given in Table 3.

#### 3.10.1. Folding Endurance

All the prepared MDFs have an acceptable folding endurance. F3 has higher folding endurance when compared with other MDFs. The results were shown in Table 3.

### 3.11. *In Vitro* Dissolution Studies

**Figure 5:**
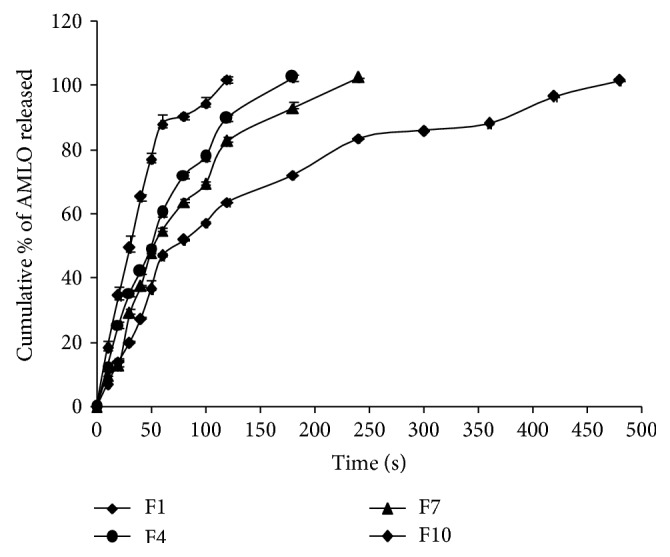
Comparative drug release profiles of F1, F4, F7, and F10.

**Figure 6:**
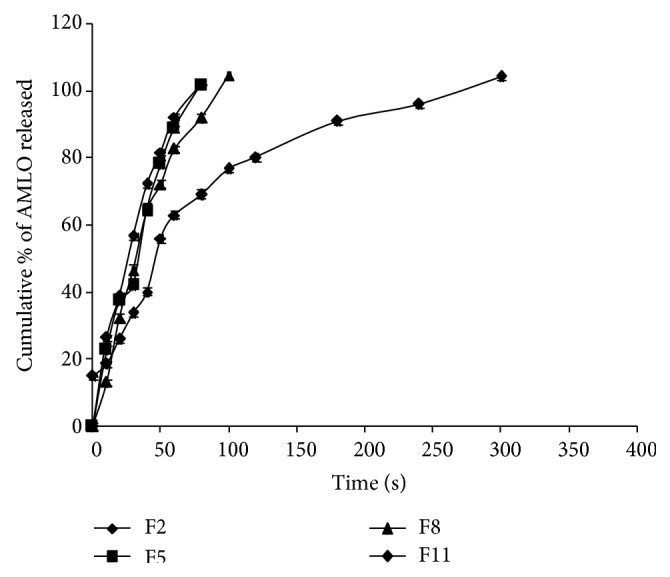
Comparative drug release profiles of F2, F5, F8, and F11.

**Figure 7:**
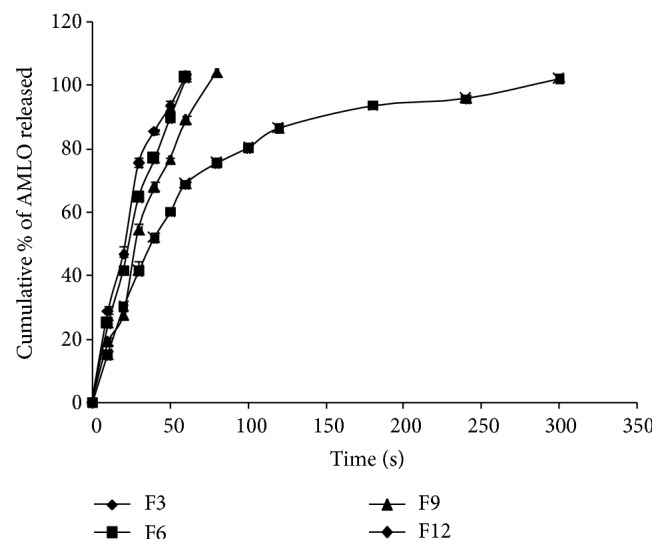
Comparative drug release profiles of F3, F6, F9, and F12.

The* in vitro* dissolution profiles of AMLO MDFs are shown in Figures 5–7. In total, 12 different formulations of AMLO were prepared using HPMC E3, HPMC E5, HPMC E15, and MC as film forming polymers with and without SLS and PVP K30.

The cumulative percent AMLO released at the end of 10 sec is 18.36 ± 0.23, 12.52 ± 1.47, 9.52 ± 1.32, and 6.96 ± 0.40 for F1, F4, F7, and F10, respectively. Complete AMLO release was obtained at 120, 180, 240, and 480 sec for F1, F4, F7, and F10, respectively, and the comparative release profile was shown in Figure 5. The AMLO release from F1 (only E3) is significantly higher when compared to F4 (only E5), F7 (only E15), and F10 (only MC). Overall, the order of percent AMLO dissolution from MDFs is F1 > F4 > F7 > F10.

Effect of solubilizing and or wetting agents on AMLO release was also tested. Both the SLS and PVP K30 were added to the formulations at 0.04% level. The cumulative percent of AMLO released at the end of 10 sec for MDFs with SLS is 26.44 ± 0.94, 22.85 ± 0.93, 13.00 ± 1.94, and 11.38 ± 2.25 for F2, F5, F8, and F11, respectively. Complete AMLO release was obtained at 80 sec for F2 and F5 and 100 sec for F8 and 360 sec for F11. The comparative release profile was shown in Figure 6.

The cumulative percent of AMLO released at the end of 10 sec for MDFs with PVP is 28.89 ± 1.38, 24.74 ± 1.42, 19.18 ± 1.15, and 14.92 ± 0.67 for F3, F6, F9, and F12, respectively. The comparative release profiles are given in Figure 7. Complete AMLO release was obtained at 60 sec for both F3 and F6, 80 sec for F9, and 300 sec for F12.

Overall, the E3 formulations (F1, F2, F3) with and without SLS and PVP gave superior dissolution properties when compared to E5 formulations (F4, F5, F6), E15 formulations (F7, F8, F9), and MC formulations (F10, F11, F12). This could be due to the low viscosity of the HPMC E3 polymer when compared to E5, E15, and MC polymers. MDFs with PVP and SLS gave superior dissolution properties when compared to MDFs without PVP and SLS. MDFs with PVP (F3, F6, F9, and F12) gave superior dissolution properties when compared to the SLS formulations (F2, F5, F8, and F11).

### 3.12. Drug Release Kinetics

To better understand the release profiles obtained with AMLO MDFs formulations, the drug release data obtained at different time points was fitted in to kinetic models such as zero order [15], first order [16], and Higuchi models [17]. The release rate constant values and correlation coefficient (*R* ^2^) values calculated from dissolution data (0–50 sec) for AMLO MDFs were given in Table 4.

When compared to F1 (only E3) the first order release rate constant “*k*” values were significantly higher for F2 and F3 containing SLS and PVP. A 1.13 and 1.53 folds increase in “*k*” values for F2 and F3 when compared to F1 was observed. Overall, the “*k*” values were in the order of F3 > F2 > F1.

When compared to F4 (only E5), “*k*” values were significantly higher for F5 and F6 containing SLS and PVP. A 2.14 and 2.71 folds increase in “*k*” values for F5 for F6 when compared to F4 was observed. When compared to F7 (only E15), “*k*” values were significantly higher for F8 and F9 containing SLS and PVP. A 2.16 and 2.49 folds increase in “*k*” values for F8 for F9 when compared to F7 was observed. When compared to F10 (only MC), “*k*” values were significantly higher for F11 and F12 containing SLS and PVP. A 1.33 and 2 folds increase in “*k*” values for F11 and 2 folds for F12 when compared to F10 was observed.

Overall, MDFs with SLS and PVP gave higher “*k*” values when compared to MDFs without SLS and PVP. MDFs with PVP gave higher “*k*” values when compared to MDFs with SLS. Among all 12 formulations the “*k*” value was significantly higher for F3. The Higuchi square root model of all the formulations showed higher correlation coefficient values (0.936–0.979), indicating diffusion as the release mechanism. The results are given in Table 4. Based on the above results, the F3 showed the highest dissolution rate and lowest* in vitro* disintegration time values as appropriate for MDFs.

## 4. Conclusion

From this investigation, it can be concluded that AMLO can be successfully formulated in to MDFs. All the MDFs prepared with HPMC E3, E5, E15, and MC as film formers possessed good physicomechanical and dissolution properties. Among the 12 formulations prepared, the F3 (7.5% w/w HPMC E3 as film former and 0.04% w/w PVP K30) gave higher* in vitro* AMLO release (102.92 ± 1.15% at the end of 60 sec). The MDFs showed no change in the homogeneity, transparency, color, and smoothness properties even at the end of the 6-month time period (25°C/65% RH) when compared to initial properties and especially no crystallization of the AMLO was observed. These results are indicative of the stability of AMLO in MDFs. The developed AMLO MDFs may provide quick onset of action with improved oral bioavailability and enhanced patient compliance and therapeutic efficacy when compared to the current marketed formulations like IR and ODTs.

## Figures and Tables

**Table 1:** Composition of different MDFs containing AMLO.

Ingredients (mg)	Formulae (5 g size)											
	F1	F2	F3	F4	F5	F6	F7	F8	F9	F10	F11	F12
AMLO	100	100	100	100	100	100	100	100	100	100	100	100
HPMC E3	375	375	375	—	—	—	—	—	—	—	—	—
HPMC E5	—	—	—	375	375	375	—	—	—	—	—	—
HPMC E15	—	—	—	—	—	—	375	375	375	—	—	—
MC	—	—	—	—	—	—	—	—	—	100	100	100
PEG 400	25	25	25	25	25	25	25	25	25	25	25	25
SLS	—	2	—	—	2	—	—	2	—	—	2	—
PVP	—	—	2	—	—	2	—	—	2	—	—	2
Water	1730	1728	1728	1730	1728	1728	1730	1728	1728	1780	1778	1778
Methanol	2750	2750	2750	2750	2750	2750	2750	2750	2750	2950	2950	2950
Pineapple flavor	10	10	10	10	10	10	10	10	10	10	10	10
Aspartame	10	10	10	10	10	10	10	10	10	10	10	10

**Table 2:** Physicomechanical properties of different AMLO MDFs∗.

Formulations	Drug content (mg/cm^2^) (*n* = 3)	Mass variation (mg)	Thickness (*µ*m) (*n* = 6)	Disintegration time (sec)	
				Drop method (*n* = 3)	Petri dish method (*n* = 3)
F1	0.911 ± 0.0046	3.40 ± 0.20	56.67 ± 5.47	19.67 ± 1.53	36.33 ± 1.15
F2	1.244 ± 0.0085	3.60 ± 0.17	68.33 ± 4.08	14.67 ± 0.58	31.33 ± 1.53
F3	1.241 ± 0.0095	3.57 ± 0.15	68.33 ± 4.08	10.33 ± 0.58	20.67 ± 0.58
F4	0.904 ± 0.0055	3.47 ± 0.21	56.67 ± 5.16	23.67 ± 0.58	45.33 ± 2.52
F5	1.240 ± 0.0134	3.77 ± 0.15	68.33 ± 0.00	21.33 ± 0.58	36.67 ± 1.53
F6	1.233 ± 0.0232	3.73 ± 0.16	68.33 ± 0.00	12.33 ± 1.15	23.33 ± 0.58
F7	0.958 ± 0.0105	3.67 ± 0.21	58.33 ± 4.08	26.33 ± 0.58	55.33 ± 0.58
F8	1.208 ± 0.0133	3.93 ± 0.06	70.00 ± 0.00	21.67 ± 0.58	49.33 ± 0.58
F9	1.095 ± 0.0391	3.97 ± 0.12	70.00 ± 0.00	17.67 ± 0.58	30.67 ± 2.08
F10	0.964 ± 0.008	3.77 ± 0.12	60.00 ± 0.00	141.67 ± 2.89	723.33 ± 5.77
F11	1.247 ± 0.049	4.10 ± 0.10	70.00 ± 0.00	62.67 ± 2.31	656.67 ± 5.77
F12	1.216 ± 0.013	4.05 ± 0.10	70.00 ± 0.00	55.00 ± 1.73	568.33 ± 2.89

^*^No change in the values after a 6-month period was observed.

**Table 3:** Physicomechanical properties of different AMLO MDFs.

Formulations	Tensile strength (N/cm^2^)	% Elongation (cm %)	Elasticity modulus (N/cm^2^)	Folding endurance
F1	2.23 ± 0.15	85.53 ± 3.60	3.38 ± 0.24	101
F2	3.26 ± 0.19	80.83 ± 3.22	2.26 ± 0.15	98
F3	2.13 ± 0.25	94.43 ± 3.66	1.28 ± 0.10	135
F4	1.90 ± 0.17	69.16 ± 3.18	2.76 ± 0.21	112
F5	2.46 ± 0.21	72.06 ± 2.73	1.61 ± 0.28	109
F6	3.40 ± 0.28	88.96 ± 3.12	2.36 ± 0.07	119
F7	2.20 ± 0.36	79.90 ± 1.35	3.32 ± 0.24	83
F8	4.53 ± 0.40	82.63 ± 1.95	2.68 ± 0.16	99
F9	2.90 ± 0.22	88.26 ± 0.96	2.16 ± 0.19	95
F10	13.16 ± 1.40	58.96 ± 2.47	19.81 ± 1.35	75
F11	8.43 ± 0.65	71.43 ± 3.40	9.87 ± 0.23	81
F12	6.80 ± 0.45	77.26 ± 2.75	10.09 ± 0.43	92

**Table 4:** Drug release kinectics data.

Formulations	DP_10_ (mean ± SD)	*R* ^2^ (first order plot)	Mean “*k*” (sec^−1^) (0–50 sec)	Higuchi constant *K*_*H*_ (sec^−1/2^)	*R* ^2^ (Higuchi plot)
F1	18.36 ± 0.23	0.977	0.0345	10.30	0.956
F2	26.44 ± 0.94	0.981	0.0391	12.16	0.977
F3	28.89 ± 1.38	0.973	0.0529	14.13	0.964
F4	12.52 ± 1.47	0.997	0.0161	8.45	0.979
F5	22.85 ± 0.93	0.961	0.0345	10.30	0.956
F6	24.74 ± 1.42	0.959	0.0437	13.72	0.950
F7	9.52 ± 1.32	0.976	0.0138	7.52	0.967
F8	13.00 ± 1.94	0.983	0.0299	10.30	0.956
F9	19.18 ± 1.15	0.971	0.0345	12.58	0.936
F10	6.96 ± 0.40	0.975	0.00691	4.90	0.961
F11	11.38 ± 2.25	0.985	0.00921	5.89	0.952
F12	14.92 ± 0.67	0.934	0.01382	5.23	0.938

DP_10_: drug percent released at 10 sec.
